# Effectiveness of Healthcare Interventions on Smoking Cessation in Adolescents in Low- and Middle-Income Countries: A Narrative Review

**DOI:** 10.7759/cureus.54051

**Published:** 2024-02-12

**Authors:** Janhvi Thakur, Sonali G Choudhari

**Affiliations:** 1 Epidemiology and Public Health, Datta Meghe Institute of Higher Education and Research, Wardha, IND; 2 Epidemiology and Public Health, Community Medicine, Jawaharlal Nehru Medical College, Datta Meghe Institute of Higher Education and Research, Wardha, IND

**Keywords:** smoking in lmic, adolescent smoking, tobacco cessation, smoking interventions, smoking cessation

## Abstract

The smoking epidemic is the greatest threat to global public health that has ever existed. Teenagers constantly perceive smoking as a way to communicate with friends and express their emotions. Adolescent smokers should be noticed immediately before the extended period of tobacco addiction and being resistant to the interventions. The aim of this study is to assess the effectiveness of healthcare interventions targeting smoking cessation in adolescents within low- and middle-income countries (LMICs) and to summarize the options, benefits, and affordability of interventions for quitting smoking that may be implemented in LMICs. The review highlights the effectiveness of various pharmacological interventions, such as nicotine replacement therapy (NRT), bupropion, nortriptyline, varenicline, and cytosine, shedding light on their respective success rates in aiding smoking cessation. Additionally, the study delves into the realm of behavioral interventions, emphasizing the significant impact of counseling, brief advice, and automated text messaging in promoting smoking cessation among adolescents. Furthermore, the review examines the influence of external factors, such as an increase in cigarette prices and changes in the smoking environment, on smoking habits. It underscores the importance of creating smoke-free areas and leveraging community involvement to enhance the effectiveness of interventions. The study also evaluates the affordability and sustainability of smoking cessation interventions, emphasizing the need for a balanced combination of pharmacotherapy and behavioral support. The advantages of quitting smoking will improve the nation’s health and boost economic productivity.

## Introduction and background

The leading cause of preventable death worldwide is the use of tobacco. Most tobacco-related fatalities occur in low- and middle-income countries (LMICs) [1]. The papers in this thematic issue discuss various challenges and successes globally in enhancing capacity, adapting, assessing, and disseminating effective treatments in LMICs. A significant and shared challenge is the integration of brief cessation counseling into healthcare practices. Despite patients considering physicians highly credible for interventions, healthcare providers, although capable of professionally assisting patients in quitting, do not consistently or effectively offer cessation services. The issue is complex; in many LMICs, physicians and other healthcare providers have elevated tobacco use rates, leading to hesitancy in supporting their patients' efforts to quit. In addition to motivational challenges, many physicians are doubtful about patients seeking their advice to quit smoking, don't see cessation as part of their professional responsibilities, and may have misconceptions about the harms of tobacco [2]. According to the World Health Organization (WHO), 22% of adolescents over 15 years old smoke tobacco regularly [3]. While the smoking prevalence among male adolescents witnessed a decline after 2011, it has plateaued over the past three years. On the other hand, the smoking rate among female adolescents experienced a decrease until 2016, followed by a subsequent rise in the last three years. This scenario underscores the pressing necessity for timely interventions aimed at fostering healthy behaviors among children and adolescents, thereby mitigating the potential risk of adverse health outcomes in later life [4].

Despite advanced technological assistance, a persistent challenge arises when evidence-based interventions crafted in high-income countries prove unsuitable for LMIC contexts without significant modification. As highlighted by Asfar et al. in 2016, behavioral cessation strategies with empirical support developed and widely implemented in the US and UK, such as self-monitoring, nicotine fading, and social support enhancement, do not find acceptance or utility in Syria. Similarly, research from Pakistan and Syria indicates that incorporating pharmacotherapy into behavioral support within "real-world" healthcare settings may not enhance cessation rates beyond what can be achieved with behavioral support alone [2]. In some LMICs like Pakistan, India, Bangladesh, and Nepal, the rate of smoking is very high among adolescents, at 26%-29% among those under 14 years old [5].

India encounters specific difficulties due to the widespread use of various forms of tobacco and the constrained resources allocated for tobacco control (International Institute for Population Sciences, 2010) [6]. Teenagers constantly perceive smoking as a way to communicate with friends and express their emotions [4]. More than 5,500 children under the age of 15 start using tobacco every day. Approximately five million Indian adolescents smoke regularly [7]. Although the estimated adult smoking rate decreased from 24.7% in 2005 to 22.2% in 2015 across 126 countries, smoking is still a crucial public health concern worldwide [8]. The World Health Organization and the Indian Ministry of Health have set up a limited number of cessation clinics in major cities; nevertheless, the capacity of these clinics falls significantly short of addressing the extent of the issue [6]. By 2030, it is estimated that tobacco consumption will result in the deaths of more than 10 million individuals each year [9]. India is predicted to have the highest increase in tobacco use among all other nations by 2030, and there will be more than 1.5 million tobacco-related deaths annually [10].

Although the use of tobacco has been rising in developing nations, little is known about various efforts to stop smoking [11]. It is necessary to introduce effective strategies for achieving a larger tobacco control community [12]. The anti-smoking campaign through radio or outside promotion had no discernible impact [5]. However, advertising campaigns, financial incentives, and legislation banning smoking in public spaces are just some of the interventions to encourage smoking cessation [13]. Similarly to high-income nations, LMICs exhibit significant disparities in tobacco use and its associated health impacts. Tobacco consumption is more prevalent among men, individuals with lower educational attainment, those with limited household wealth, residents of rural areas, and older age groups in LMICs [14]. Media messages on anti-smoking, particularly those broadcast on television, were successful in discouraging teenagers aged 12-13 from smoking [5].

This review emphasizes various options of pharmacotherapy and behavioral interventions that may be effectively utilized as monotherapy or in combination to treat tobacco addiction among adolescent smokers in LMICs.

## Review

Methodology

**Figure 1:**
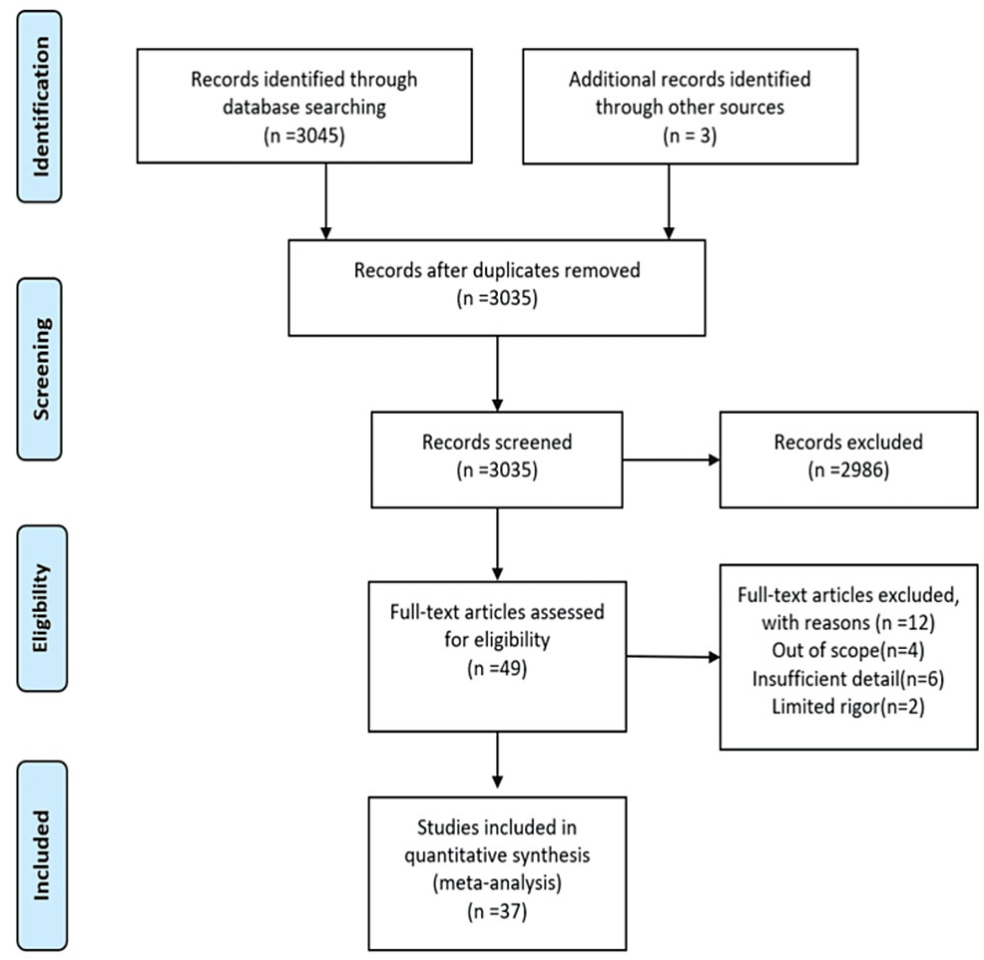
Inclusion and exclusion criteria of the study

This narrative review focuses on the effectiveness of interventions to stop smoking among adolescents from LMICs. Relevant articles were selected after a thorough search using PubMed, Google Scholar, and the Cochrane Library database. The total number of records discovered through database searching was N = 3045. Additional records (N = 3) were found using different sources. After removing duplicates, there were 3,035 records. The records excluded were 2,986. From this full-text article evaluated for eligibility, N = 49. Of these articles, 37 are used as references, and 12 are excluded with reasons. The following key searches were used: “smoking cessation, smoking in LMICs, effects of smoking intervention, smoking interventions, adolescent smoking, tobacco use”, (smoking cessation [Title/Abstract]) AND (intervention [Title/Abstract]), (effects of smoking [Title/Abstract]) AND (cessation [Title/Abstract]), (smoking cessation [Title/Abstract]) AND (intervention [Title/Abstract]) AND (low-income countries [Title/Abstract]) smoking cessation, interventions on smoking, adolescents smoking. The records that were included were qualitative and quantitative research conducted in LMICs, tobacco cessation, adolescent smoking, and studies published from 2008 until now. The records excluded were studies published in languages other than the English language, research conducted in high-income countries, age groups other than adolescents, and studies with insufficient information regarding the effectiveness of smoking cessation interventions. The data was taken from the articles that were published. We checked reference lists of retrieved studies for relevant articles and separately evaluated all the prospective studies we pointed out as a result of the search strategy. The inclusion and exclusion criteria of the study are given in Figure 1.

Discussion

Decades of investigations carried out in LMICs provide evidence-based interventions for smoking cessation [15]. The majority (76% of published studies) stated that smoking cessation interventions were successful in helping adolescents quit [15]. Cessation by the current sufferer is the only realistic method to avoid the death toll related to tobacco globally before 2050 [2]. According to a study, by increasing the 10% cigarette tax, the odds of a teen quitting smoking would rise by 10% [16]. Numerous countries offer healthcare interventions that encourage and assist in quitting smoking, as supported by lots of studies. In addition to a variety of drugs, such as nicotine replacement therapy (NRT), varenicline, bupropion, nortriptyline, and cytosine, the approaches also include behavioral counseling and direct assistance from healthcare professionals to be more successful in triggering the quit-smoking decision [13]. Cost-effective cessation strategies are required for implementation in resource-constrained environments like India, with a focus on widespread dissemination [5]. It is crucial to identify the initiatives or programs that adolescents value the most. In a country with limited resources, there is a need for evidence demonstrating the effectiveness of any anti-tobacco initiatives before they are put into practice. Additionally, it is essential to grasp the socio-cultural context specific to this region, as it can impact the smoking behavior of youth and consequently shape their attitudes and reactions toward anti-smoking initiatives [4].

Interventions for smoking cessation

Pharmacotherapy Interventions

To treat substance use disorders holistically, pharmacotherapy refers to the use of pharmaceuticals in conjunction with behavioral therapies and counseling [15]. Some pharmacological interventions include the following:

Nicotine replacement therapy (NRT): This therapy aims to reduce withdrawal symptoms associated with stopping smoking by replacing the nicotine from cigarettes. This is available as skin patches that deliver nicotine slowly, chewing gum, nasal spray, inhalers, and lozenges or tablets, all of which deliver nicotine to the brain more quickly than skin patches but less rapidly than smoking cigarettes [16]. Not a single product on the market can provide such high nicotine dosages as fast as cigarettes. A cigarette typically contains 1 to 3 milligrams of nicotine, and a smoker who smokes twenty cigarettes a day will absorb 20 to 40 milligrams of nicotine daily [17]. This intervention raises six- to 12-month uninterrupted abstention rates by 6%, significantly compared to a placebo when provided to those who smoke 15 or more cigarettes daily [10].

Bupropion: The FDA has approved bupropion for adult smokers with tobacco use disorders. Because of its action as a norepinephrine and dopamine reuptake inhibitor, it is most frequently used as an antidepressant; nevertheless, it also functions as an antagonist of the nicotinic receptor. By inhibiting the effects of nicotine and raising dopamine and norepinephrine levels in the brain, bupropion's distinct mode of action helps lessen the symptoms of withdrawal [15]. Numerous studies have demonstrated that bupropion increased the uninterrupted abstinence rate of around one year by 7% compared to a placebo in daily smokers [10].

Nortriptyline: It is one of the tricyclic antidepressants. The typical dosage for smoking cessation is 75 to 100 mg/day for 12 to 14 weeks. Smokers who smoke at least 15 cigarettes per day have been reported to increase 10% of a six- to 12-month uninterrupted abstinence rate [10].

Varenicline: The most successful pharmacotherapy for quitting smoking is varenicline. It is a partial agonist that binds with great affinity to the nicotinic acetylcholine receptor, which comprises beta-2 and alpha-4 subunits. A typical treatment plan is 1 mg daily, starting one week before the planned quit date, followed by 11 weeks of taking 2 mg daily. Nausea and disturbed sleep are minor adverse effects that have been recorded [18]. This drug raises the abstinence rate of smoking for six- to 12-month periods by 15% [10].

Cytisine: It was the pioneer of stop-smoking aids and has been prescribed in Eastern Europe for over four decades. Nausea is a frequent mild side effect of cytisine but does not cause smokers to stop their medication abruptly [8]. A typical treatment course lasts four weeks, starting one week before the designated quit date and involving a gradually reduced dosage. It has been used for more than 40 years in Eastern Europe and was the first drug ever licensed as a smoking cessation aid [10].

Behavioral interventions: For the past 20 years, behavioral interventions have dominated adolescent smoking cessation [19]. Counseling alone might raise the likelihood of quitting smoking by 40% to 80%. This indicated that seven out of every 100 smokers successfully quit for at least six months while receiving the same transient support as the control groups. It would be expected that 10 to 12 out of every 100 people who receive counseling will succeed [20].

Brief advice: Brief opportunistic advice includes discussing the side effects of smoking and advising smokers to give it up [10]. In Delhi's slum neighborhoods, non-medical healthcare providers found a rise in six-month biochemically confirmed uninterrupted abstention rates of 95% after giving brief advice on quitting smoking door-to-door [10]. A study found that the effect of simple advice on cessation rates was negligible. The short advice may boost quitting by 1%-3%, with an unattended quit rate of 2-3%. Even though there is a slight advantage to more thorough interventions compared to brief advice, additional components have a negligible impact [21].

 *Increase in Cigarette Prices*

Several studies have persistently indicated that the higher cost of cigarettes reduces smoking habits. The increasing price of cigarettes might lower smoking rates, increase smoking cessation, and delay the initiation of smoking [22].

 Automated Text Messaging

Automated text messaging focuses on motivational messages, tips for dealing with cravings, and behavioral distractions when necessary. Given the high rate of mobile phone ownership in LMICs, this approach could attract broad participation. The messages were similar to the information in person-to-person behavioral support. The automated text elevated the six- to 12-month abstention rate by 4% [10].

Changes in the smoking environment

As a result of interventions, smoking and quitting rates improved, which increased the smoking-free areas. A better understanding of the adverse effects of tobacco is necessary to gain public support for disclosing the high usage rates of tobacco. A study on tobacco use among Aboriginal youth revealed that smoke-free territories, more physical activities, and access to recreational opportunities would motivate them to avoid smoking [23].

The effectiveness and affordability of smoking cessation interventions

The interventions for smoking cessation have varied effectiveness, but overall, the combination of pharmacotherapy and behavioral intervention is more favorable [10]. The affordability should also be considered for the sustainability of implementing the interventions (Table 1). A narrative review also demonstrated the effectiveness of combining brief advice and pharmacotherapy [24]. Many people do not trust the adverse effects of smoking promoted on the cigarette packet or media before facing the personal experience [5]. Helping smokers to quit is more effective by promoting other tobacco preventive measures like product labeling and community involvement [13]. The effectiveness and affordability of the smoking cessation interventions have been mentioned in Table 1.

**Table 1:** The effectiveness and affordability of the smoking cessation interventions

Intervention	Effectiveness	Affordability
Nicotine replacement therapy [8, 14]	Help moderate- or heavy smokers when combined with at least some behavioral support. The best outcome is when a transdermic patch is combined with a quicker-acting form.	Universally affordable
Bupropion [10]	At least as successful as single-form NRT when it helps moderately heavy or heavy smokers quit, mainly when used in conjunction with behavioral support.	Affordable in middle- and high-income countries
Nortriptyline [10]	Effective when combined with behavioral interventions for moderate or heavy smokers. Patients report occasional mild side effects, most notably dry mouth, but not enough to compromise its efficacy.	Universally affordable
Varenicline [8, 15]	More efficient than bupropion and single-form NRT and equally successful as combination NRT when combined with behavioral support on moderate and heavy smokers.	Affordable in middle- and high-income countries
Cytisine [8, 15, 16]	Has been reported to be more effective compared to NRT without the combination of behavioral interventions.	Universally affordable
Brief advice [8, 13, 17]	Considering a 2 to 3% unassisted quit rate, a brief advice intervention increases the quit rate by 2% when done for around 20 minutes.	Universally affordable
Increase in cigarette prices [22]	Effective even for heavy smokers. The quantity of cigarettes smoked daily decreased in response to price rises.	Not mentioned
Changes in smoking environment [23]	Effective by distracting the smoking lifestyle if applied thoroughly to modifying the activities and surroundings.	Not mentioned
Automated text messaging [10]	Effective if the information is focused on the tested methods with clear benefits.	Universally affordable

Smoking mainly triggers the occurrence of non-communicable diseases, which is considered to be a contributing factor in 16% of all non-communicable disease-related deaths [25]. The neighborhood impacts whether people decide to smoke or not [26]. Higher odds of quitting or trying to quit have been associated with living in rural areas, having more education, and having more wealth [27]. In Bihar, India, the Tobacco Free Teachers-Tobacco Free Society (TFT-TFS) program involved teachers and health educators in promoting and enacting anti-tobacco school policies. The program took seven months, from September to March. The goal of the intervention was to encourage students and teachers to quit smoking and be aware of smoking-related health problems. This study demonstrated the psychological effects of anti-tobacco messages on adolescents [28].

The Global Adult Tobacco Survey (GATS) has been conducted to monitor the control of tobacco use in nations with high smoking rates, which comprehends the tobacco dependence in LMICs [29]. According to surveys, one-third of smokers made an annual attempt to stop, and three out of four smokers wanted to stop. Only 1-3% of people succeeded when they tried by themselves [30]. A biomedical study demonstrated that smoking tobacco and secondhand smoke contributed to early death and a more comprehensive range of non-communicable diseases, such as heart disease, cerebrovascular disease, and lung cancer [21,24]. In contrast, smokers' health conditions gradually improve as they quit smoking (Table 1). Helping smokers quit is sophisticated in LMICs due to scarce resources and a lack of accessible and cost-effective pharmacotherapy [6]. Another obstacle could be the tendency of national governments to fund initiatives, such as research, to prevent future issues rather than allocating limited funds to solve urgent, pressing health concerns [31]. The health advantages of smoking cessation have been mentioned in Table 2 [7].

**Table 2:** Health advantages of smoking cessation

Time duration after quitting	Health benefits
24 hours	Lungs begin to expel mucus and other smoke-related debris
48 hours	Eliminate the systemic carbon monoxide.
72 hours	Lighter breathing
Two to 12 weeks	Better circulation
Three to nine months	The lung capacity rises by up to 10%
One year	Difficulties and symptoms of coughing, wheezing, and breathing problems improve
10 years	The risk of developing lung cancer drops by half
15 years	The chance of a heart attack decreases

Smokers who consulted a physician had a higher possibility of quitting smoking than those who did not. Peer pressure and the inability to halt smoking cravings were reported as the main barriers to quitting [32]. Consequently, the first worldwide agreement to control tobacco, the WHO Framework Convention on Tobacco Control (WHO FCTC), encouraged healthcare professionals to promote regular screening for smokers and guidance to give up smoking [33]. The WHO FCTC Article 14 recommendations state that countries that would like to deal with the reduction of smokers have to ensure widespread access to cessation support at a reasonable price and encourage attempts at quitting smoking by improving or building sustainable infrastructure [34]. Article 6 of the WHO FCTC specifically mandates nations to enact tax measures and price policies to lower tobacco smoking. Interventions that uplift tobacco production costs lower tobacco use in high-income and LMICs [10]. The FCTC and the overall efficacy of tobacco control are mainly due to the expansion and influence of a global tobacco control movement [35].

Considering the significantly lower smoking prevalence in high-income nations, tobacco cessation studies are necessary in LMICs. For instance, between 1990 and 2015, Australia's male smoking prevalence improved at an annualized rate of 2.2%. Smoking rates continue to rise in LMICs, for example, in Bangladesh, which reported an increase in male smoking cases by 0.3% annually from 1990-2015 [1]. Monitoring the situation among adolescents in every nation is crucial, even if data on those 15 years of age and older is used to measure the worldwide targets for reduced tobacco use [36]. While this is the present trend, it is not guaranteed that the burden of tobacco use in LMICs will decrease. Future results can be changed by ongoing studies and focused action to lower tobacco use, including strategies for quitting combined with robust tobacco control laws [37].

Low- and middle-income countries often underutilize cessation assistance. Advocates for tobacco control in these nations should focus on developing strategies to encourage the utilization of cessation support, aiming to improve quit rates. Some suggested research directions include tailoring interventions for specific populations, long-term effectiveness and relapse prevention, digital health interventions, economic and policy interventions, biomarkers, and monitoring technologies.

## Conclusions

In high-income countries, a more robust impact on adolescent smoking cessation is observed when a combination of approaches, including smoking-related education, nicotine replacement therapy, automated text messaging anti-smoking campaigns, tobacco price hikes, bans on public smoking, and smoking cessation programs, is employed rather than relying on a single or isolated method, and these interventions are found to be globally affordable. This study revealed various smoking cessation interventions that may be implemented in LMICs. Based on their effectiveness and affordability, NRT, nortriptyline, cytisine, brief professional advice, and automated text messaging are potentially utilized in LMICs. In conclusion, smoking cessation interventions have proven to be effective tools in the global effort to reduce smoking prevalence and improve public health. A combination of pharmacological and behavioral approaches, tailored to individual needs, offers a comprehensive strategy for achieving successful smoking cessation outcomes. Continued efforts in research, public awareness, and policy implementation are essential to further enhance the effectiveness of these interventions and create a tobacco-free future.

## References

[1] Kumar N, Janmohamed K, Jiang J (2021). Tobacco cessation in low- to middle-income countries: A scoping review of randomized controlled trials. Addict Behav.

[2] Ward KD (2016). Tobacco intervention research in low- and middle-income countries: lessons learned and future directions. J Smok Cessat.

[3] Gonzálvez MT, Espada JP, Orgilés M, Sussman S (2017). Two-year effects of a classroom-based smoking prevention and cessation intervention program. Eur Addict Res.

[4] Choi Y, Lee CM, Cho B, Lee ES, Oh SW, Lee N, Yun JM (2021). Behavioral interventions for smoking cessation among adolescents: a rapid review and meta-analysis for the Korea Preventive Services Task Force. Osong Public Health Res Perspect.

[5] Rao S, Aslam SK, Zaheer S, Shafique K (2014). Anti-smoking initiatives and current smoking among 19,643 adolescents in South Asia: findings from the Global Youth Tobacco Survey. Harm Reduct J.

[6] Aghi M, Nagler E, Lando H, Pednekar M, Gupta P, Sorensen G (2016). Training lay interventionists to support tobacco cessation among teachers in India. Int J Health Promot Educ.

[7] Van Schayck OC, Williams S, Barchilon V (2017). Treating tobacco dependence: guidance for primary care on life-saving interventions. Position statement of the IPCRG. NPJ Prim Care Respir Med.

[8] Lin Y, Dlodlo RA, Shu Q (2019). Outcomes of a smoking cessation intervention at follow-up after 5 years among tuberculosis patients in China. Tob Induc Dis.

[9] Ghose S, Sardar A, Shiva S, Mullan BE, Datta SS (2019). Perception of tobacco use in young adults in urban India: a qualitative exploration with relevant health policy analysis. Ecancermedicalscience.

[10] Sarkar BK, West R, Arora M, Ahluwalia JS, Reddy KS, Shahab L (2017). Effectiveness of a brief community outreach tobacco cessation intervention in India: a cluster-randomised controlled trial (the BABEX Trial). Thorax.

[11] Wang L, Jin Y, Lu B, Ferketich AK (2016). A cross-country study of smoking cessation assistance utilization in 16 low and middle income countries: data from the Global Adult Tobacco Survey (2008-2012). Nicotine Tob Res.

[12] Warner KE (2013). An endgame for tobacco?. Tob Control.

[13] West R, Raw M, McNeill A (2015). Health-care interventions to promote and assist tobacco cessation: a review of efficacy, effectiveness and affordability for use in national guideline development. Addiction.

[14] Shankar A, Parascandola M, Sakthivel P, Kaur J, Saini D, Jayaraj NP (2022). Advancing tobacco cessation in LMICs. Curr Oncol.

[15] Akanbi MO, Carroll AJ, Achenbach C (2019). The efficacy of smoking cessation interventions in low- and middle-income countries: a systematic review and meta-analysis. Addiction.

[16] Oncken CA, Dietz PM, Tong VT (2010). Prenatal tobacco prevention and cessation interventions for women in low- and middle-income countries. Acta Obstet Gynecol Scand.

[17] Squeglia LM, Fadus MC, McClure EA, Tomko RL, Gray KM (2019). Pharmacological treatment of youth substance use disorders. J Child Adolesc Psychopharmacol.

[18] Stead LF, Perera R, Bullen C, Mant D, Lancaster T (2008). Nicotine replacement therapy for smoking cessation. Cochrane Database Syst Rev.

[19] Hartmann-Boyce J, Chepkin SC, Ye W, Bullen C, Lancaster T (2018). Nicotine replacement therapy versus control for smoking cessation. Cochrane Database Syst Rev.

[20] Courtney RJ, McRobbie H, Tutka P (2021). Effect of cytisine vs varenicline on smoking cessation: a randomized clinical trial. JAMA.

[21] Schepis TS, Rao U (2008). Smoking cessation for adolescents: a review of pharmacological and psychosocial treatments. Curr Drug Abuse Rev.

[22] Lancaster T, Stead LF (2017). Individual behavioural counselling for smoking cessation. Cochrane Database Syst Rev.

[23] Stead LF, Buitrago D, Preciado N, Sanchez G, Hartmann-Boyce J, Lancaster T (2013). Physician advice for smoking cessation. Cochrane Database Syst Rev.

[24] Cavazos-Rehg PA, Krauss MJ, Spitznagel EL (2014). Differential effects of cigarette price changes on adult smoking behaviours. Tob Control.

[25] Minichiello A, Lefkowitz AR, Firestone M, Smylie JK, Schwartz R (2016). Effective strategies to reduce commercial tobacco use in Indigenous communities globally: a systematic review. BMC Public Health.

[26] Zvolska K, Pankova A, Nohavova I (2020). A narrative review of facilitators and barriers to smoking cessation and tobacco-dependence treatment in patients with tuberculosis in low- and middle-income countries. Tob Induc Dis.

[27] Swain SK, Chatterjee K, Basannar DR (2021). Efficacy of group intervention on tobacco cessation among male employees in health-care setting: a randomized controlled trial. Med J Armed Forces India.

[28] Chow CK, Corsi DJ, Gilmore AB (2017). Tobacco control environment: cross-sectional survey of policy implementation, social unacceptability, knowledge of tobacco health harms and relationship to quit ratio in 17 low-income, middle-income and high-income countries. BMJ Open.

[29] Shang C, Chaloupka F, Kostova D (2014). Who quits? An overview of quitters in low- and middle-income countries. Nicotine Tob Res.

[30] Nargis N, Yong HH, Driezen P (2019). Socioeconomic patterns of smoking cessation behavior in low and middle-income countries: emerging evidence from the Global Adult Tobacco Surveys and International Tobacco Control Surveys. PLoS One.

[31] Siddiqi K, Khan A, Ahmad M, Shafiq-ur-Rehman Shafiq-ur-Rehman (2010). An intervention to stop smoking among patients suspected of TB--evaluation of an integrated approach. BMC Public Health.

[32] Hamann SL, Mock J, Hense S, Charoenca N, Kungskulniti N (2012). Building tobacco control research in Thailand: meeting the need for innovative change in Asia. Health Res Policy Syst.

[33] Owusu-Dabo E, Lewis S, McNeill A, Gilmore A, Britton J (2011). Support for smoke-free policy, and awareness of tobacco health effects and use of smoking cessation therapy in a developing country. BMC Public Health.

[34] Owusu D, Wang KS, Quinn M, Aibangbee J, John RM, Mamudu HM (2019). Health care provider intervention and utilization of cessation assistance in 12 low- and middle-income countries. Nicotine Tob Res.

[35] Ahluwalia IB, Tripp AL, Dean AK, Mbulo L, Arrazola RA, Twentyman E, King BA (2021). Tobacco smoking cessation and quitline use among adults aged ≥15 years in 31 countries: findings from the Global Adult Tobacco Survey. Am J Prev Med.

[36] Reddy KS, Yadav A, Arora M, Nazar GP (2012). Integrating tobacco control into health and development agendas. Tob Control.

[37] World Health Organization (2019). WHO global report on trends in prevalence of tobacco use 2000-2025. Who Global Report on Trends in Prevalence of Tobacco Use 2000-2025.

